# Effect of Verapamil, an L-Type Calcium Channel Inhibitor, on Caveolin-3 Expression in Septic Mouse Hearts

**DOI:** 10.1155/2021/6667074

**Published:** 2021-04-08

**Authors:** Bruna A. C. Rattis, Ana C. Freitas, Jordana F. Oliveira, João L. A. Calandrini-Lima, Maria J. Figueiredo, Danilo F. Soave, Simone G. Ramos, Mara R. N. Celes

**Affiliations:** ^1^Department of Pathology, Faculty of Medicine of Ribeirão Preto, University of São Paulo, Ribeirão Preto, São Paulo, Brazil; ^2^Department of Bioscience and Technology, Institute of Tropical Pathology and Public Health, Federal University of Goias, Goiânia, Goias, Brazil; ^3^Department of Morphofunctional, Faculty of Medicine of Goianesia (FAMEGO), University of Rio Verde (UniRV), Goianesia, Goias, Brazil

## Abstract

Sepsis-induced myocardial dysfunction considerably increases mortality risk in patients with sepsis. Previous studies from our group have shown that sepsis alters the expression of structural proteins in cardiac cells, resulting in cardiomyocyte degeneration and impaired communication between cardiac cells. Caveolin-3 (CAV3) is a structural protein present in caveolae, located in the membrane of cardiac muscle cells, which regulates physiological processes such as calcium homeostasis. In sepsis, there is a disruption of calcium homeostasis, which increases the concentration of intracellular calcium, which can lead to the activation of potent cellular enzymes/proteases which cause severe cellular injury and death. The purpose of the present study was to test the hypotheses that sepsis induces CAV3 overexpression in the heart, and the regulation of L-type calcium channels directly relates to the regulation of CAV3 expression. Severe sepsis increases the expression of CAV3 in the heart, as immunostaining in our study showed CAV3 presence in the cardiomyocyte membrane and cytoplasm, in comparison with our control groups (without sepsis) that showed CAV3 presence predominantly in the plasma membrane. The administration of verapamil, an L-type calcium channel inhibitor, resulted in a decrease in mortality rates of septic mice. This effect was accompanied by a reduction in the expression of CAV3 and attenuation of cardiac lesions in septic mice treated with verapamil. Our results indicate that CAV3 has a vital role in cardiac dysfunction development in sepsis and that the regulation of L-type calcium channels may be related to its expression.

## 1. Introduction

Sepsis is a potentially fatal organ dysfunction, characterized by an unregulated response of a host to an infection [1]. Despite significant advances in diagnosis and therapeutic approaches in recent years, sepsis remains the major cause of death in intensive care units (ICUs). A robust study from Rudd et al. showed that there are about 48.9 million cases of sepsis each year, causing 11 million deaths worldwide [2]. A study estimated that about 200,000 deaths are caused by sepsis in Brazilian ICUs each year [3]. Sepsis patients are often affected by sepsis-induced myocardial dysfunction (SIMD), which is associated with worse prognoses and higher mortality rates when compared to patients with sepsis without SIMD [4–9]. These patients may present global biventricular dysfunction (systolic or diastolic) with reduced contractility, left ventricular dilation, and decreased response to resuscitation with fluids and catecholamines [9].

Previous results from our research group demonstrated that structural changes in cardiac cells elucidate the physiopathology of SIMD [10]. During experimental sepsis, loss and reduction of structural protein expressions were implicated with compromised functioning of cardiac cells [10]. The reduction of connexin-43 and N-cadherin resulted in the loss of structural integrity of intercalated discs, hindering communication between myocardial cells [11]. Subsequently, cardiomyocyte degeneration and lysis of actin and myosin filaments, all caused by sepsis, were associated with reduced expression of dystrophin [12].

Dystrophin proteins act as critical components of the dystrophin-glycoprotein complex (DGC), establishing connections between the intracellular cardiac contractile machinery and the extracellular matrix. Furthermore, DGC performs three essential basic functions: stabilization of the membrane during contraction cycles, transduction of contractile force, and the organization of membrane specializations [13]. In addition to dystrophin, evidence indicates that CAV3 is localized to the sarcolemma, where it associates with the DGC [14, 15]. CAV3 is part of the caveolin group (caveolins-1, 2, and 3); caveolins-1 (CAV1) and 2 are expressed in most cell types, including adipocytes, smooth muscle cells, endothelial cells, epithelial cells, and fibroblasts whereas caveolin-3 is expressed in striated and cardiac muscle tissue [16]. These groups are concentrated in regions rich in cholesterol and sphingolipids called lipids rafts, forming the caveolae [16, 17]. The caveolae are vesicular invaginations of the plasma membrane, responsible for regulating endocytosis, exocytosis, signal transduction, mechanoprotection, cholesterol, and calcium homeostasis [16, 18, 19].

Caveolae are associated with several ion channels in cardiomyocytes, such as long-lived and voltage-dependent L-type Ca^2+^ channels (LTCCs) [19, 20]. CAV3 is colocated with the *α*1 isoform of LTCCs in cardiomyocytes in the Cav*β* region [19, 21, 22]. Caveolae can modulate the process of excitation-contraction of cardiac cells, regulating the calcium transient and response to *β*-adrenergic stimulation. In addition, the loss of caveolae decreases the amplitude of the transient [Ca^2+^]_i_, reducing the contraction [19, 23, 24].

The loss of calcium homeostasis is harmful to the cell. The intracellular increase of this ion activates proteases, nucleases, and ATPases that lead to cell death. In vitro studies have shown a significant increase in the concentration of free intracellular calcium [Ca^2+^]_i_ in cardiomyocytes exposed to the serum of septic mice [25]. The role of CAV3 in septic cardiomyocytes and its relationship to calcium are still unclear. Thus, the present study is aimed at evaluating the expression of caveolin-3 in the heart of septic mice associated with verapamil treatment, an L-type calcium channel antagonist.

## 2. Materials and Methods

### 2.1. Experimental Animals

Male C57BL/6 mice, weighing 22–24 g, were maintained at ambient room temperature (22 ± 2°C) under a 12/12-hour light-dark cycle. They were housed at the Animal Facility of the Department of Pathology of the Faculty of Medicine of Ribeirão Preto and given standard mouse feed and water *ad libitum*. The animal protocol was approved by the Committee on Animal Research of the Faculty of Medicine of Ribeirão Preto, University of São Paulo, Brazil (Protocol no. 083/2012). All efforts were made to minimize animal suffering.

### 2.2. Polymicrobial Sepsis (Cecal Ligation and Puncture (CLP) Model)

A modified CLP model was used to induce polymicrobial sepsis [25]. The mice were quickly anesthetized with 2.0% isoflurane, vaporized in medical oxygen (O_2_), via a face mask. The abdomen was shaved, and a midline incision was performed. The cecum was isolated and ligated with 6–0 silk thread below the ileocecal valve without causing bowel obstruction. The cecum was then punctured with an 18-gauge needle to induce severe septic injury (SSI). Bowel content was gently extruded through the puncture, and the cecum was then replaced to its original position. The abdomen was then sutured. Sham-operated animals (controls) underwent the same procedures, except for cecal ligation and puncturing. To prevent dehydration, all mice received subcutaneous doses of saline (50 mL/kg of body weight) immediately and 12 hours after the surgical procedure. For pain relief, sodium dipyrone solution (10 mg/100 g body weight, i.p.) was administered at the start of the surgery and 6-12 hours after surgery. Mice were monitored daily for signs of disease, such as piloerection, hunched gait, lethargy, and eye discharge. Mice displaying severe signs of distress (labored breathing, nonresponsiveness to cage tapping, failure of grooming, and severe eye discharge) were humanely euthanized by injecting a mixture of ketamine (90–120 mg/kg) and xylazine (10 mg/kg), followed by cervical dislocation.

### 2.3. Experimental Groups and Drug

For the experiments, male C57BL/6 mice were arbitrarily allocated into four groups: (1) sham, (2) SSI, (3) sham+verapamil (SH+VP), and (4) SSI+verapamil (SSI+VP). The verapamil hydrochloride (5 mg/kg body weight, Sigma-Aldrich Co., St. Louis, USA) was diluted in sterile 0.9% NaCl saline (100 *μ*L total volume/animal) and injected intraperitoneally (i.p.) two hours after CLP surgery (SSI+VP) or the sham operation (SH+VP). Untreated control (sham) and untreated septic mice (SSI) received an equivalent volume of saline. The survival rates were monitored every 12 hours for five days after surgery using 10 animals per group (sham, SSI, SH+VP, and SSI+VP, *n* = 10 per group).

### 2.4. Histopathology

For the histopathology analyses, mice were euthanized with 100 *μ*L of a 10 : 1 mixture of ketamine (90–120 mg/kg) and xylazine (10 mg/kg), respectively. The thoracic cavity was opened, and the heart was removed 24 hours after surgery (*n* = 6 animals/group SSI/SSI+VP and *n* = 4 animals/group sham/SH+VP). Hearts were longitudinally sectioned into two halves; one-half of the heart was fixed in phosphate-buffered 10% formalin and embedded in Historesin (Leica Instruments, Heidelberg, Germany) for high-resolution light microscopy. The 2 *μ*m thick sections were stained with toluidine blue, and left ventricles were analyzed. Another half of the hearts were frozen at -80°C for the immunoblotting procedure.

### 2.5. Immunohistochemistry

For the immunostaining of CAV3, immunohistochemistry was performed. The slides were deparaffinized in an oven (55°C for 30 minutes) and a xylene bath. The cuts were then hydrated in decreasing alcohol concentrations of 100%, 90%, and 70%. Subsequently, the slides were placed in warm distilled water and underwent antigenic recovery in citrate buffer (pH 6.0) at 95°C. Consecutively, the slides went to the inactivation stage of endogenous peroxidase with 3% hydrogen peroxide solution (H_2_O_2_) for three minutes. The slides were then incubated with 2% BSA for 25 minutes. After, sections were incubated with primary antibody (anti-caveolin-3; BD Transduction Laboratories) diluted at the concentration of 1 : 1000 in blocking buffer overnight (18 hours) at 4°C, in a humidified chamber. Subsequently, the sections were incubated with biotinylated secondary anti-mouse antibody (LSAB®+ Kit, K0675, Dako Corporation, Carpinteria, United States) for 20 minutes and then with streptavidin peroxidase solution for 20 minutes (LSAB®+ Kit, K0675, Dako Corporation). The reaction was developed from the chromogenic solution of diaminobenzidine (DAB) (3,3′-diaminobenzidine, Sigma) and prepared with 1 mL of substrate (hydrogen peroxide (H_2_O_2_) 3%) for one minute. The cuts were washed briefly in distilled water. In this process, the slides were counterstained for 30 seconds in hematoxylin and placed in a container for washing with running water for eight minutes. The cuts underwent dehydration in alcohol of 70%, 95%, and 100% and in xylene. Finally, the slides were mounted with the coverslip using the Entellan mounting medium. A 0.01 M phosphate-buffered saline solution (PBS) with pH 7.2-7.4 was used to wash the cuts.

### 2.6. Western Blotting

To determine the amount of CAV3 in the hearts of sham (*n* = 4), SSI (*n* = 6), SH+VP (*n* = 4), and SSI+VP (*n* = 6) mice, homogenates of left ventricles were submitted to immunoblotting 6, 12, and 24 hours after the CLP or sham procedure. Hearts of mice were homogenized in the modified RIPA buffer lysis (Tris HCl 0.05°M (pH°7.4); NaCl 0.15°M; EDTA 0.001°M (pH°8.0); SDS 0.1%) supplemented with a protease inhibitor cocktail (Sigma-Aldrich) and the phosphatase inhibitors (Na_3_VO_4_ 0.001°M; NaF 0.025°M; Na_4_P_2_O_7_ 0.0005°M). This buffer does not separate cytosolic protein from plasma membrane protein. Equal concentrations (50°*μ*g/well) of total proteins (homogenate) were resolved on 10% SDS-Page gels and transferred to a PVDF membrane (Amersham Pharmacia Biotech, Amersham, UK). The membranes were blocked with 5% albumin for two hours and incubated overnight at 4°C with the primary antibodies: anti-caveolin-3 (mouse monoclonal antibody, 1 : 10000; BD Transduction Laboratories) and anti-GAPDH (rabbit monoclonal antibody, 1 : 1000; Cell Signaling Technology). Then, the blots were washed and incubated with HRP-conjugated secondary antibodies for one hour at room temperature. Membranes were washed, developed using ECL (Amersham Pharmacia Biotech), and viewed with ChemiDoc XRS (BioRad). Image analysis was performed using the public domain ImageJ program (developed at the National Institutes of Health and available at http://rbs.info.nih.gov/nih-image/) with the “Gel Analysis” function. Analysis results are represented by the values of each band; each value is proportional to the integrated density value (IDV) of the specific band, which corresponds to the arbitrary unit (AU). GAPDH was used to determine equivalent loading conditions.

### 2.7. Statistical Analysis

Data were analyzed using the GraphPad Prism 5 statistics program (GraphPad Software Inc., San Diego, United States). Data were expressed as means ± standard deviation (S.D.). Statistically significant differences between groups for western blot analysis were measured by one-way analysis of variance (ANOVA) followed by post hoc Tukey's multiple comparison test (parametric data). Statistical analysis of survival curves was performed using the Kaplan-Meier with a Mantel-Cox (log-rank) test. *P* < 0.05 was considered statistically significant. All *P* values are demonstrated in the graphics.

## 3. Results

### 3.1. Sepsis Survival Rates

**Figure 1:**
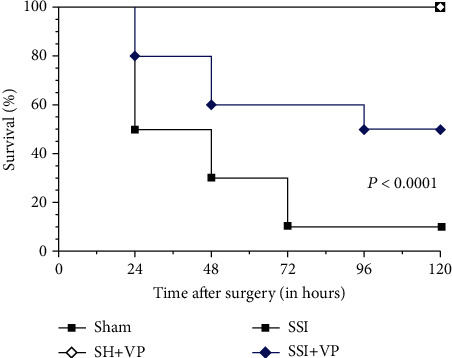
Survival curve of the mice subjected to cecal ligation and puncture (CLP) sepsis. Groups of 10 mice were submitted to sham operation (sham) or severe septic injury (SSI) and were treated with verapamil (SH+VP, SSI+VP). The survival rate was determined daily up to 120 hours after surgery. Statistical analysis was performed using the Kaplan-Meier with a Mantel-Cox (log-rank) test. Survival curves obtained with verapamil treatment (SSI+VP) were significantly different (*P* < 0.001) as compared to the sepsis group (SSI).

Figure 1 shows the survival rate of mice submitted to the sham operation (sham) and SSI until 120 hours after surgery. The sham (sham) and sham-treated (SH+VP) animals showed full recovery from anesthesia and maintained 100% survival until the end of the observation. The SSI mice showed a 50% survival rate 24 hours after injury, decreasing to a 10% survival rate 72 hours after cecal puncture. Rates then remained steady until 120 hours after surgery. In contrast, the treated septic mice (SSI+VP) showed a survival rate of around 80% in 24 hours, decreasing to 50% at 96 hours. Rates then remained stable until the end of observation at 120 hours.

### 3.2. Effect of Verapamil Administration on Cardiac Lesions

**Figure 2:**
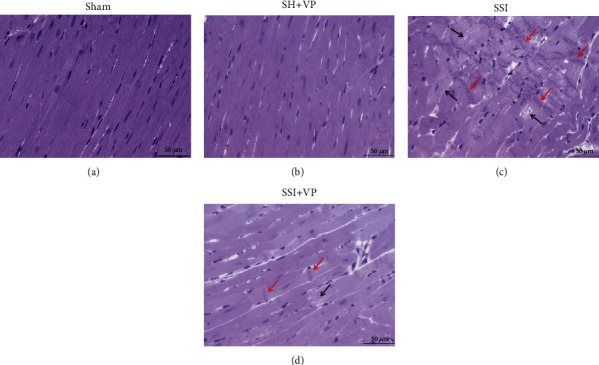
Histopathology of myocardial tissue from mice subjected to cecal ligation and puncture (CLP) sepsis. The sham-operated mice (sham and SH+VP) showed no changes (a, b). The SSI group (c) had evident disorientation of the myofibrils with the formation of contracture bands (red arrows) and myocytolysis (black arrows) as compared to the SSI+VP group (d), 24 hours after surgery. Scale bars indicate 50 *μ*m.

Histopathological analyses of the heart showed that severe sepsis resulted in extensive lesions in the myocardium (Figure 2). After 24 hours of sepsis induction, the cardiac tissue septic group (SSI) presented regions of myofibril disorientation with the formation of contraction bands, necrosis, and an apparent rupture of the sarcolemma. However, septic mice treated with verapamil (SSI+VP) had more preserved cardiomyocytes and less cellular changes than the untreated septic group (SSI). The sham-operated mice (sham and SH+VP) showed no changes.

### 3.3. Effect of Verapamil Administration on Caveolin-3 Distribution in the Heart

**Figure 3:**
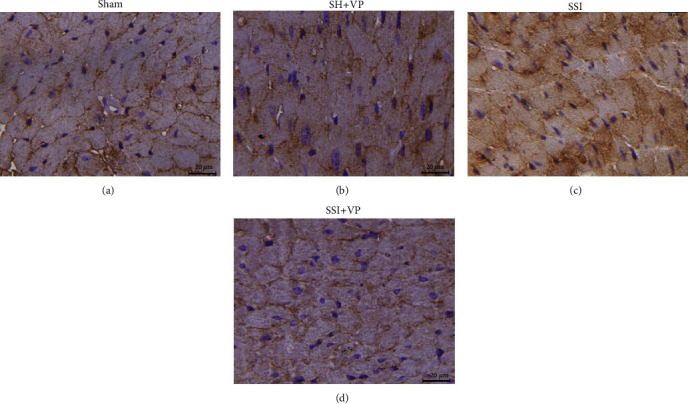
Immunolocalization of caveolin-3 in cardiac tissue 24 hours after sepsis induction. (a, b) Show the immunostaining of CAV3 on cardiomyocytes (sham and SH+VP) bounded by the plasma membrane. (c) Represents the scattered staining of CAV3 in the cytoplasm and cell membrane of septic cardiomyocytes (SSI group). (d) The septic mice treated with verapamil (SSI+VP) showed immunostaining of CAV3 more related to that observed in the control groups (sham and SH+VP). Scale bars indicate 50 *μ*m.

Figure 3 shows the distribution of CAV3 in the cardiac cells 24 hours after sepsis induction. In the cardiomyocytes of the control groups (sham and SH+VP), CAV3 was delimited in the plasma membrane of the cells. The untreated septic group (SSI) presented immunostaining of CAV3 scattered throughout the cytoplasm (not membrane-bound fraction) and plasma membrane of the heart cells. In contrast, when treated with verapamil, the septic mice (SSI+VP group) showed immunostaining of CAV3 closer to that observed in the control groups (sham and SH+VP).

### 3.4. Effects of Verapamil Administration on Caveolin-3 Expression in the Heart

**Figure 4:**
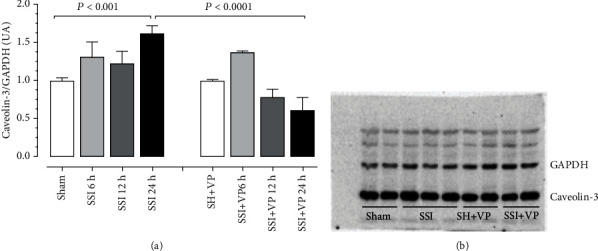
Western blot analysis of CAV3. (a) The amounts of CAV3 in the sham (*n* = 4) and SH+VP (*n* = 4) groups were measured 24 hours after sham operation. The amounts of CAV3 in the SSI (*n* = 6) and SSI+VP (*n* = 6) groups were measured 6, 12, and 24 hours after CLP surgery and expressed in arbitrary units (AUs). GAPDH was used to determine equivalent loading conditions. Note that the expression of CAV3 was significantly increased in the SSI group 24 hours after CLP as compared to SSI+VP and sham group mice. Statistical analysis was performed by one-way ANOVA followed by Tukey's test. Data were expressed as the mean ± SD; *P* < 0.0001 (SSI vs. SSI+VP) and *P* < 0.001 (SSI vs. sham). (b) The autoradiograph resulting from western blot analysis of representative protein levels for CAV3 and GAPDH of mouse hearts, subjected to sham operation (sham, SH+VP) or sepsis induction (SSI, SSI+VP) 24 hours after surgery.

Figure 4 shows the quantitative analysis of CAV3 protein levels in the myocardium of controls (sham, SH+VP) and animals subjected to severe sepsis (SSI) and treated with verapamil (SSI+VP) 6, 12, and 24 hours after surgery. The results showed a significant increase in the levels of CAV3 expression only 24 hours after the severe sepsis induction (SSI) when compared to the values observed in the hearts of the control group (sham). For the slight increase in the levels of CAV3, 6 and 12 hours after CLP surgery, there was no statistical difference among the groups. Additionally, septic mice treated with verapamil (SSI+VP) showed significantly reduced levels of CAV3 24 hours after CLP surgery when compared to untreated septic animals (SSI).

## 4. Discussion

In this study, we demonstrated for the first time that CAV3 is overexpressed in the hearts of septic mice, and the treatment with verapamil influenced the reduction of CAV3 in septic mouse hearts. Additionally, reduced expression of CAV3 led to a reduction of sepsis-induced cardiac injuries and a decreased mortality rate.

Septic patients frequently develop hypocalcemia [26]. However, calcium is essential in several physiological processes, such as excitation-contraction of cardiac cells. Thus, parenteral calcium administration could potentially generate positive results in these hypocalcemic patients [27]. Calcium supplementation in septic patients and animals has been shown to increase mortality rates and lead to organ failure [28, 29]. Interestingly, intracellular calcium concentrations are increased in sepsis; this has been associated with pathophysiological changes [30]. The displacement of calcium into the cells may be largely responsible for hypocalcemia. Although parenteral calcium administration appears to be the solution, it can contribute to organ dysfunction [26, 29].

A previous study from our research group showed an increase in [Ca^2+^]_i_ in cultured neonatal cardiomyocytes treated with septic animal serum [31]. Calcium overload by CLP has also been demonstrated in the heart, brain, liver, and spleen cells of septic rats [32]. The hypotheses for this increase in [Ca^2+^]_i_ involve failures in the channels that regulate the entry of Ca^2+^, microruptures in the plasma membrane, and the excessive release of Ca^2+^ by the sarcoplasmic reticulum [31, 33, 34].

However, one hypocalcemia hypothesis suggests that sepsis-induced failures in calcium channels cause an increased influx of Ca^2+^ into cells [32]. This hypothesis is supported by the fact that the administration of a calcium channel blocker results in a better prognosis and a reduction in mortality rates of septic patients [35]. These data corroborate with experimental findings; the administration of verapamil in septic animals resulted in a reduction of mortality, attenuation of cardiac lesions, reduction of intracellular calcium concentration, and attenuation of hypocalcemia [31, 32, 36]. The data from the present study also supports this hypothesis, as septic animals treated with verapamil survived longer than septic animals without treatment.

The increase in [Ca^2+^]_i_ activates proteases inside the cell, such as calpain. In sepsis, calpain expression in cardiomyocytes increases, with a concomitant reduction in dystrophin-glycoprotein complex (DGC) proteins [37]. The disturbance of this complex, the consequent reduction in dystrophin, and the contraction process make the cell more susceptible to mechanical stress in the plasma membrane, resulting in its rupture [38, 39]. The consequences of dystrophin reduction can be seen in Duchenne Muscular Dystrophy (DMD). DMD patients can develop cardiomyopathy with cardiac cell loss. This leads to greater vulnerability to pressure overload and can result in dilated cardiomyopathy [40]. Experimental models of DMD have shown that the loss of dystrophin causes a progressive increase in the expression of CAV3 in the plasma membrane, cytoplasm, and caveolae in muscle cells [41]. Consistent with these results, we observed that CAV3 was overexpressed in mouse hearts with sepsis, demonstrating its presence in the plasma membrane and cytoplasm through immunostaining. As previously demonstrated, this occurred even with the activation of proteases such as calpain. This indicates that CAV3 does not undergo the process of degradation mediated by calpain, as observed with dystrophin [37].

A study using the lung of septic mice (induced by the CLP model) demonstrated a reduction of CAV1, which was reported as a host cytoprotective factor to regulate the number of available caveolae that can be used by pathogens as an escape mechanism from lysosomal degradation [42]. Surprisingly, human lung endothelial cells challenged with LPS exhibited a concentration- and time-dependent increased expression of CAV1 mRNA and protein. This effect has been found to be dependent on NF-*κ*B activation and thereby contributes to the mechanism of microvascular permeability in sepsis [43].

The cause of CAV3 overexpression in sepsis is still unknown. Studies that induced overexpression of CAV3 in transgenic mice showed severe cardiomyocyte degeneration with reduced cardiac function, in addition to skeletal muscle damage and negative regulation of DGC; such findings are similar to those found in DMD [44, 45]. However, CAV3 knockout mice developed progressive cardiomyopathy and an incorrect DGC complex location [46]. It is surprising to see reduced CAV3 expressions in pathological cardiac conditions, such as myocardial infarction, heart failure, and hypertrophy [20, 47]. Our study demonstrates that cardiac changes induced by sepsis provide a different response regarding the expression of CAV3, as septic animals showed a significant increase in CAV3 levels.

There are strong indications that CAV3 regulates calcium homeostasis in cardiac cells, an important relationship in maintaining cellular physiology. One study showed the absence of slow Ca^2+^ waves in cells absent from CAV3, and this also occurred when the interaction of CAV3 with G protein was interrupted [48]. On the other hand, the induced overexpression of CAV3 interrupted the hypertrophic signaling caused by pressure overload through the inhibition of the type T calcium channel current and the suppression of the Ca^2+^-dependent calcineurin-NFAT pathway [20].

## 5. Conclusions

Our results indicate that sepsis leads to increased expression of CAV3 in the heart, and the treatment with verapamil can directly or indirectly modulate its expression resulting in a reduction of mortality rates and cardiac injuries.

## Data Availability

The data used to support the findings of this study are available from the corresponding authors upon request.

## References

[1] Singer M., Deutschman C. S., Seymour C. W. (2016). The third international consensus definitions for sepsis and septic shock (sepsis-3). *JAMA*.

[2] Rudd K. E., Johnson S. C., Agesa K. M. (2020). Global, regional, and national sepsis incidence and mortality, 1990-2017: analysis for the Global Burden of Disease Study. *Lancet*.

[3] Machado F. R., Cavalcanti A. B., Bozza F. A. (2017). The epidemiology of sepsis in Brazilian intensive care units (the Sepsis PREvalence Assessment Database, SPREAD): an observational study. *The Lancet Infectious Diseases*.

[4] Burton A., Waisbren M. D. (1951). Bacteremia due to Gram-negative bacilli other than the Salmonella. *A.M.A. Archives of Internal Medicine*.

[5] Parrillo J. E. (1990). Septic shock in humans. *Annals of Internal Medicine*.

[6] Merx M. W., Weber C. (2007). Sepsis and the heart. *Circulation*.

[7] de Souza Dantas V. C., Costa E. L. V. (2015). A look at the diastolic function in severe sepsis and septic shock. *Revista Brasileira de terapia intensiva*.

[8] Wei Cheng M. M., Long Y., Wang H., Wen Han M. M., Zhang J., Cui N. (2019). Role of the mTOR signalling pathway in human sepsis-induced myocardial dysfunction. *Canadian Journal of Cardiology*.

[9] Heureux M. L., Sternberg M., Brath L., Turlington J., Kashiouris M. G. (2020). Sepsis-induced cardiomyopathy: a comprehensive review. *Current Cardiology Reports*.

[10] Celes M. R. N., Prado C. M., Rossi M. A. (2013). Sepsis: going to the heart of the matter. *Pathobiology*.

[11] Celes M. R. N., Torres-Dueñas D., Alves-Filho J. C., Duarte D. B., Cunha F. Q., Rossi M. A. (2007). Reduction of gap and adherens junction proteins and intercalated disc structural remodeling in the hearts of mice submitted to severe cecal ligation and puncture sepsis. *Critical Care Medicine*.

[12] Celes M. R. N., Torres-Dueñas D., Malvestio L. M. (2010). Disruption of sarcolemmal dystrophin and _*β*_ -dystroglycan may be a potential mechanism for myocardial dysfunction in severe sepsis. *Laboratory Investigation*.

[13] Lapidos K. A., Kakkar R., McNally E. M. (2004). The dystrophin glycoprotein complex: signaling strength and integrity for the sarcolemma. *Circulation Research*.

[14] Van Deurs B., Roepstorff K., Hommelgaard A. M., Sandvig K. (2003). Caveolae: anchored, multifunctional platforms in the lipid ocean. *Trends in Cell Biology*.

[15] Davies K. E., Nowak K. J. (2006). Molecular mechanisms of muscular dystrophies: old and new players. *Nature Reviews. Molecular Cell Biology*.

[16] Thomas C. M., Smart E. J. (2008). Caveolae structure and function. *Journal of Cellular and Molecular Medicine*.

[17] Busija A. R., Patel H. H., Insel P. A. (2017). Caveolins and cavins in the trafficking, maturation, and degradation of caveolae: implications for cell physiology. *American Journal of Physiology. Cell Physiology*.

[18] Anderson R. G. W. (1993). Potocytosis of small molecules and ions by caveolae. *Trends in Cell Biology*.

[19] Balijepalli K. (2008). Caveolae, ion channels and cardiac arrhythmias. *Progress in Biophysics and Molecular Biology*.

[20] Markandeya Y. S., Phelan L. J., Woon M. T. (2015). Caveolin-3 overexpression attenuates cardiac hypertrophy via inhibition of T-type Ca^2+^ current modulated by protein kinase C*α* in cardiomyocytes. *Journal of Biological Chemistry*.

[21] Abriel H., Rougier J.-S., Jalife J. (2015). Ion channel macromolecular complexes in cardiomyocytes: roles in sudden cardiac death. *Circulation Research*.

[22] Rougier J. S., Abriel H. (2016). Cardiac voltage-gated calcium channel macromolecular complexes. *Biochimica et Biophysica Acta (BBA) - Molecular Cell Research*.

[23] Calaghan S., White E. (2006). Caveolae modulate excitation-contraction coupling and *β*2- adrenergic signalling in adult rat ventricular myocytes. *Cardiovascular Research*.

[24] Barbuti A., Terragni B., Brioschi C., DiFrancesco D. (2007). Localization of f-channels to caveolae mediates specific *β*_2_-adrenergic receptor modulation of rate in sinoatrial myocytes. *Journal of Molecular and Cellular Cardiology*.

[25] Wichterman K. A., Baue A. E., Chaudry I. H. (1980). Sepsis and septic shock--a review of laboratory models and a proposal. *The Journal of Surgical Research*.

[26] Steele T., Kolamunnage-Dona R., Downey C., Toh C. H., Welters I. (2013). Assessment and clinical course of hypocalcemia in critical illness. *Critical Care*.

[27] Forsythe R. M., Wessel C. B., Billiar T. R., Angus D. C., Rosengart M. R. (2008). Parenteral calcium for intensive care unit patients. *Cochrane Database of Systematic Reviews*.

[28] MALCOLM D. I. A. N. A. S., ZALOGA G. A. R. Y. P., HOLADAV J. O. H. N. W. (1989). Calcium administration increases the mortality of endotoxic shock in rats. *Critical Care Medicine*.

[29] Collage R. D., Howell G. M., Zhang X. (2013). Calcium supplementation during sepsis exacerbates organ failure and mortality via calcium/calmodulin-dependent protein kinase kinase signaling. *Critical Care Medicine*.

[30] Song S. K., Karl I. E., Ackerman J. J., Hotchkiss R. S. (1993). Increased intracellular Ca2+: a critical link in the pathophysiology of sepsis?. *Proceedings of the National Academy of Sciences of the United States of America*.

[31] Celes M. R. N., Malvestio L. M., Suadicani S. O. (2013). Disruption of calcium homeostasis in cardiomyocytes underlies cardiac structural and functional changes in severe sepsis. *PLoS One*.

[32] He W., Huang L., Luo H., Zang Y., An Y., Zhang W. (2020). Hypocalcemia in sepsis: analysis of the subcellular distribution of Ca2+ in septic rats and LPS/TNF-*α*-treated HUVECs. *Journal of Infection in Developing Countries*.

[33] Fanchaouy M., Polakova E., Jung C., Ogrodnik J., Shirokova N., Niggli E. (2009). Pathways of abnormal stress-induced Ca^2+^ influx into dystrophic _mdx_ cardiomyocytes. *Cell Calcium*.

[34] Sepúlveda M., Gonano L. A., Viotti M. (2017). Calcium/calmodulin protein kinase II-dependent ryanodine receptor phosphorylation mediates cardiac contractile dysfunction associated with sepsis. *Critical Care Medicine*.

[35] Lee C. C., Lee M. T. G., Lee W. C. (2017). Preadmission use of calcium channel blocking agents is associated with improved outcomes in patients with sepsis. *Critical Care Medicine*.

[36] Wyska E. (2009). Pretreatment with R(+)-verapamil significantly reduces mortality and cytokine expression in murine model of septic shock. *International Immunopharmacology*.

[37] Freitas A. C. S., Figueiredo M. J., Campos E. C. (2016). Activation of both the calpain and ubiquitin-proteasome systems contributes to septic cardiomyopathy through dystrophin loss/disruption and mTOR inhibition. *PLoS One*.

[38] Andrews N. W., Almeida P. E., Corrotte M. (2014). Damage control: Cellular mechanisms of plasma membrane repair. *Trends in Cell Biology*.

[39] Meyers T. A., Townsend D. W. (2019). Cardiac pathophysiology and the future of cardiac therapies in duchenne muscular dystrophy. *International Journal of Molecular Sciences*.

[40] Tayeb M. T. (2010). Deletion mutations in Duchenne muscular dystrophy (DMD) in Western Saudi children. *Saudi Journal of Biological Sciences*.

[41] Repetto S., Bado M., Broda P. (1999). Increased number of caveolae and caveolin-3 overexpression in Duchenne muscular dystrophy. *Biochemical and Biophysical Research Communications*.

[42] Kataki A., Karagiannidis I., Memos N. (2018). Host’s endogenous caveolin-1 expression is downregulated in the lung during sepsis to promote cytoprotection. *Shock*.

[43] Tiruppathi C., Shimizu J., Miyawaki-Shimizu K. (2008). Role of NF-*κ*B-dependent Caveolin-1 Expression in the Mechanism of Increased Endothelial Permeability Induced by Lipopolysaccharide. *Journal of Biological Chemistry*.

[44] Galbiati F., Volonte D., Chu J. B. (2000). Transgenic overexpression of caveolin-3 in skeletal muscle fibers induces a Duchenne-like muscular dystrophy phenotype. *Proceedings of the National Academy of Sciences of the United States of America*.

[45] Aravamudan B., Volonte D., Ramani R. (2003). Transgenic overexpression of caveolin-3 in the heart induces a cardiomyopathic phenotype. *Human Molecular Genetics*.

[46] Woodman S. E., Park D. S., Cohen A. W. (2002). Caveolin-3 Knock-out Mice Develop a Progressive Cardiomyopathy and Show Hyperactivation of the p42/44 MAPK Cascade. *Journal of Biological Chemistry*.

[47] Feiner E. C., Chung P., Jasmin J. F. (2011). Left ventricular dysfunction in murine models of heart failure and in failing human heart is associated with a selective decrease in the expression of caveolin-3. *Journal of Biological Chemistry*.

[48] Guo Y., Golebiewska U., Scarlata S. (2011). Modulation of Ca^2+^ Activity in Cardiomyocytes through Caveolae-G _*α*_ _q_ Interactions. *Biophysical Journal*.

